# Exploring preliminary dietary intake results using a novel dietary assessment tool with pregnant participants enrolled in a birth cohort

**DOI:** 10.1186/s13104-024-06697-9

**Published:** 2024-02-01

**Authors:** Sara Santarossa, Ashley Redding, Mackenzie Connell, Karissa Kao, Laura Susick, Jean M. Kerver

**Affiliations:** 1 Department of Public Health Sciences, Henry Ford Health, 1 Ford Place, Detroit, MI USA; 2 Department of Obstetrics, Gynecology and Reproductive Biology, College of Human Medicine, Michigan State University, East Lansing, MI USA; 3 Department of Pediatrics and Human Development, College of Human Medicine, Michigan State University, East Lansing, MI USA; 4 Epidemiology and Biostatistics, Michigan State University, East Lansing, MI USA

**Keywords:** Dietary assessment, Pregnancy, Diet quality, Nutrition

## Abstract

**Objective:**

We aimed to describe preliminary dietary intake results using DietID^™^ for dietary assessment during pregnancy. A sub-sample of participants in the Research Enterprise to Advance Children’s Health (REACH) prospective birth cohort from Detroit, MI received a unique web link to complete the DietID^™^ assessment multiple times during pregnancy. We present results for the first dietary assessment completed during pregnancy by each participant. DietID^™^ uses an image-based algorithm to estimate nutrient intake, dietary patterns, and diet quality and provides immediate results to participants. Descriptive statistics were used to summarize participant characteristics, nutrient intakes, dietary patterns, diet quality, and participant-rated accuracy of individual dietary assessment results. Differences in diet parameters were assessed by participant race with an independent t-test.

**Results:**

Participants (*n* = 84) identified as majority Black (*n* = 47; 56%), reflective of the source population. Mean (SD) maternal age and gestational age at dietary assessment were 32 (5.6) years and 14.3 (4.8) weeks, respectively. Mean dietary quality, as reported in the DietID^™^ data output as the Healthy Eating Index (HEI), was 68 (range 12–98; higher scores indicate higher diet quality) and varied significantly between Black (mean [SD] 61 [23]) and White (mean [SD] 81 [19]) race (*p* < 0.01). Mean participant-rated accuracy of individual dietary assessment results was high at 87% on a scale of 0-100% (“not quite right” to “perfect”; range 47–100%).

**Supplementary Information:**

The online version contains supplementary material available at 10.1186/s13104-024-06697-9.

## Introduction

Nutrient intake during pregnancy is critical for fetal development and has been associated with a wide variety of long-term health outcomes for both mother and child [1–3], (e.g., iron and iodine are critical for offspring neurocognitive development) [4]. Recent data from a large United States (US) cohort indicated that dietary intake of pregnant persons fell short of recommendations with the highest risk for inadequate intakes observed among non-White participants for many nutrients (e.g., vitamins A, E, B-6, folate, calcium) [5, 6]. Assessing dietary intake during pregnancy is a common goal of many epidemiological research studies [7, 8], but entails significant participant burden [1–3]. Minimizing research participant burden is important for obtaining accurate data and retaining participants in longitudinal studies.

While it has limitations, it is generally accepted that the gold standard method for estimating dietary intake is the multiple-pass 24-hr dietary recall (3 days– 2 week days and 1 weekend day) [9]. Food frequency questionnaires (FFQs), are also well accepted and often used in large studies [9]. Both 24-hr dietary recalls and FFQs are memory-based and may impose a large time burden for research participants and staff. Digital and web-based dietary assessments are more cost and time-effective and can enhance completion rates compared with traditional pen-paper dietary assessment methods [10], although limitations include the need for a computer or mobile device, Internet connectivity, and familiarity with the software [11–13]. Recently, use of image-based dietary records have increased [14, 15], with tools that rely less upon participant literacy and memory [16]. Image-based assessment methods have been shown to be more enjoyable for participants and less burdensome than FFQs, thus contributing to increased completion rates [17].

DietID™ is a novel dietary assessment tool that assesses dietary intake through Diet Quality Photo Navigation (DQPN®), a patented image-based algorithm that provides quick estimates of dietary patterns based on a series of food images [18–20]. Individual nutrient intakes and diet quality are estimated from the dietary patterns and provided to users including clinicians where it has been shown to be easy to administer in a hospital setting [21]. Initial validation results [18, 22–24] show that this tool requires a similar level of recall as the FFQ-based tools but produces results more comparable to the Automated Self-Administered 24-hour Dietary Assessment (ASA-24) method which is a free web-based tool [25]. When compared to the ASA-24 and FFQ methods, DietID™ has been shown to rapidly assess dietary intake while maintaining a high level of accuracy and reliability [22, 23]. Therefore, the purpose of this study was to assess preliminary dietary intake results using this novel platform, DietID™, during pregnancy.

## Materials and methods

### Study population

Data are from the ongoing prospective birth cohort, Research Enterprise to Advance Children’s Health (REACH). REACH has the broad goal of understanding various risk factors for outcomes such as childhood asthma and allergy and aims to enroll 3,000 maternal-child pairs from a health system in Detroit, MI (Henry Ford Health [HFH]). Recruitment for REACH began in January 2021 and is ongoing. This manuscript is based on a data-lock (i.e., a ‘snapshot’ of a database at a particular point in time while the study is in progress) including data from the cohort inception through February 2022. Reporting preliminary data before the cohort is closed is a common practice encouraged by NIH and other funders to ensure dissemination of results in a timely manner for maximum public health impact. At the time of the data pull for the current manuscript, pregnant persons that fit the REACH study’s eligibility criteria (described below) were identified (*n* = 1,405) through the HFH electronic medical records (EMR) system and *n* = 190 enrolled in REACH. EMR data shows that the obstetric population served by HFH in southeast Michigan is ethnically (18% Hispanic) and racially (20% Black, 2% Asian) diverse.

Potential REACH participants are recruited early in their pregnancy (before 20 weeks gestation). Eligible REACH participants are at least 18 years old and have received prenatal care at one of HFH’s 28 obstetrics clinics and plan to deliver at one of 4 HFH hospitals. There are no exclusion criteria for comorbid conditions or prior medical history; thus cohort participants are not selected for high-risk conditions and better represent the general obstetric population. Only one baby per pregnant person and pregnancy can be enrolled into the study (e.g., only 1 baby out of a set of twins can be enrolled).

The REACH cohort protocol includes biospecimen ascertainment and survey data collection. Data is also obtained from the EMR using scheduled data queries including contact information, gestational age, and estimated delivery date. The HFH Institutional Review board approved all study protocols. Interested and eligible participants provided informed consent via REDCap (Vanderbilt University, Nashville, TN) [26, 27].

### Dietary intake assessment

**Fig. 1:**
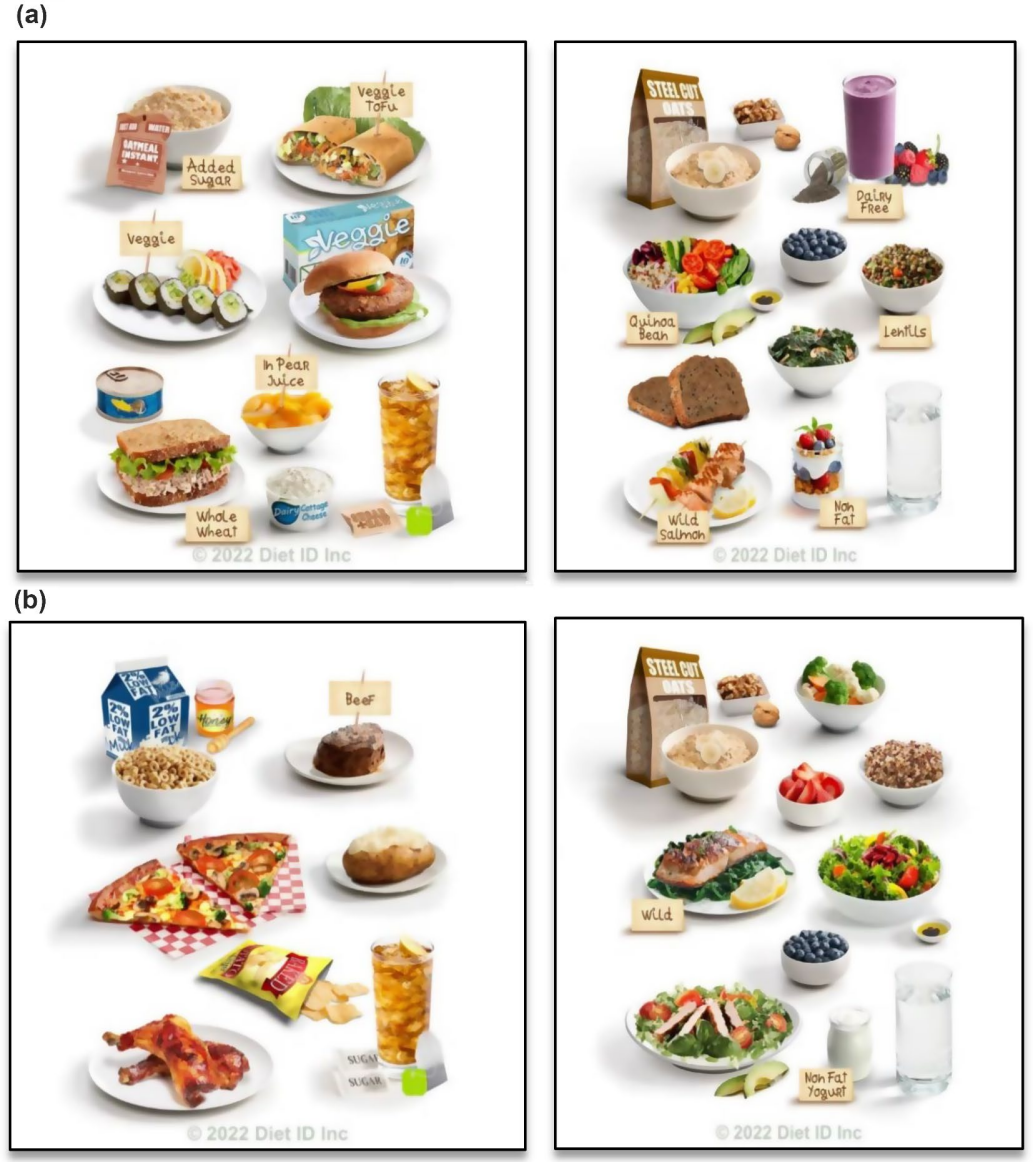
**a** Composite Images of the Flexitarian Dietary Pattern. **b** Composite Images of the Standard American Dietary Pattern. *Note* DietID™ Vignettes (i.e., Diet Quality Photo Navigation (DQPN) 1-day composite images). **a** The left image depicts a 2000-calorie standardized 1-day menu for the Flexitarian dietary pattern, Diet Quality Tier = 7 (i.e., average Diet Quality in current sample). The right image depicts a 2000-calorie standardized 1-day menu for the Flexitarian dietary pattern, Diet Quality Tier = 10 (i.e., ideal Diet Quality). *Source* Diet ID, Inc.; with permission from Diet ID, Inc. **b** The left image depicts a 2000-calorie standardized 1-day menu for Standard American dietary pattern, Diet Quality Tier = 5 (i.e., average Diet Quality in current sample).The right image depicts a 2000-calorie standardized 1-day menu for Standard American dietary pattern, Diet Quality Tier = 10 (i.e., ideal Diet Quality). *Source* Diet ID, Inc.; with permission from Diet ID, Inc

REACH participants receive a web link via email with a unique code at each dietary assessment time point (early, mid, and late pregnancy and 1-month postpartum). The early pregnancy code is sent 1-week after participants enroll into REACH (up to 20 weeks gestational age in the dataset reported here), mid and late are sent at 28- and 36-weeks gestation, respectively, and the codes do not expire. For the current study we are using the data from the first timepoint during pregnancy which the dietary assessment was completed. The link directs participants to the DietID™ survey instrument that uses DQPN® to assess respondents’ dietary patterns [18–20]. DQPN® presents a series of composite images of established dietary patterns (see Fig. 1a, b for examples, and Additional File 1 for more detailed information), asking users to select the image that most accurately resembles their self-assessed, recent, dietary intake (described below) [18–20]. For the current study, we are using a dietary intake reference period that is inclusive of the last 30 days for each data collection point. Each image selection refines the dietary assessment and participants choose between images until the “best possible fit” is achieved, and a dietary pattern is identified [18]. Participant completion time has ranged from 1 to 2 min [18–20, 23].

Detailed methodology can be obtained from the DietID™ website [19, 20], but briefly, the dietary intake output includes diet type (i.e., pattern), the Healthy Eating Index (HEI) as a commonly used measure of overall diet quality derived by comparing nutrient intakes to the US Dietary Guidelines [28], food group intake, and over 100 macro- and micro-nutrients and food components. Diet ID™ was developed using dietary data extracted from the National Health and Nutrition Examination Survey (NHANES), as well as a comprehensive review of food intake surveys and epidemiological research to determine estimates of dietary patterns, portion sizes, and eating frequencies of adults in the US [18–20]. Dietary patterns are categorized as one of 23 diet types, based on their nutrient composition [20]. Diet quality is stratified among all diet types into low to high quality tiers or levels. DietID™ derives its diet quality scores (1-to-10 scale) using the simple HEI scoring algorithm method, an index (0-to-100 scale) that measures diet quality and accounts for 13 components (food groups and nutrients) that correlate with the 2015–2020 Dietary Guidelines for Americans [29, 30]. Diet ID™ has been found to be effective in estimating diet quality, as well as nutrients and bioactive compounds associated with fruit and vegetable consumption. Figure 1 illustrates examples of the Flexitarian and Standard American dietary patterns at two different quality tiers, respectively.

The REACH team worked with DietID™ to make appropriate modifications for data collection in a pregnant population. This included modifying the reference standards for dietary recommendations to include pregnancy and lactation. Specifically, an option was added for users to choose pregnant or lactating in addition to entering their age and sex, which allows for the software to compare participant intake to the appropriate Dietary Reference Intakes (DRI) for their individual characteristics and life stage. Additionally, the research team chose specific nutrients, particular to pregnancy (folic acid, calcium, Vitamin D), to highlight for feedback provided to participants (electronically via DietID™ and the participant’s “Diet Profile”). At the end of each assessment, feedback is provided showing participants their “Diet Profile” which includes the participant’s diet type, diet quality score, an estimate of average daily calories, and estimates of selected macro- and micronutrient intakes. This is an optional feature that the research team selected to engage participants who are often interested in receiving their own results from the variety of data collected. Next, participants were asked to rate the accuracy of their dietary assessment results via a sliding scale of 0-100%, with the 0% anchor reading “not quite right” and the 100% anchor reading “perfect.”

### Data analysis

Data for this analysis are from 2/2021 to 3/2022 and include the first dietary assessment during pregnancy for each participant. Of the 190 pregnant persons enrolled in REACH, 88 (46%) had completed at least one dietary assessment during pregnancy (early, mid, or late timepoints). Participants with incomplete data entry (*n* = 3) or postpartum dietary assessment completion (*n* = 1) were excluded. The final data set included 84 pregnant participants. Maternal demographic characteristics including age, marital status, race, and gestational age were obtained from the EMR and were combined with DietID™ data output files. Descriptive statistics on selected macro- and micronutrients, diet patterns, dietary quality, and user accuracy rating were computed. An independent samples t-test was conducted to compare means of the HEI and selected nutrient intakes between Black and White race groups. Nutrient intakes were selected a priori for presentation and included macronutrients and micronutrients important during pregnancy and commonly lacking in diets of US pregnant persons [5, 31]. Data were analyzed using Microsoft Excel (Version 2108) and IBM SPSS Statistics for Windows (Version 26).

## Results

Most participants self-identified as Black (56%), with 33% White, 5% Asian, and 6% reporting other or declining. Marital status was single (54%), married (37%), living with significant other or another arrangement (10%). Maternal demographic characteristics include a mean (SD) age of 32 (5.6) years. Mean gestational age at first dietary assessment was 14 weeks, 5 days, with a median at 12 weeks, 5 days; the range was 7 weeks, 4 days to 31 weeks, 5 days. On average, participants were logged into DietID™ for 4.7 min which includes completing the screening questions (i.e., do you eat meat), the survey, and viewing results.

Table 1 describes intake of selected nutrients during pregnancy alongside dietary recommendations for pregnancy, where available. Notably, mean intakes of sodium were higher, and the mean intakes of Vitamin D and total folate were lower than recommended amounts for pregnancy.

**Table 1:** Intake of selected nutrients during pregnancy alongside dietary recommendations for pregnancy according to DietID™ in the prospective birth cohort, research enterprise to advance children’s health (REACH) (*n* = 84 pregnant persons)

Nutrient	Average intake (mean (SD))	Recommended dietary reference intakes (DRI)
Total energy (kcal)	2,193 (342)	–
**Macronutrients**		
Carbohydrates (g)	272.6 (62.6)	(45–65% of daily calories)
Total sugars (g)	92.9 (30.8)	ND
Added sugars (g)	42.4 (42.0)	ND
Dietary fiber (g)	32.9 (18.5)	[28]
Protein (g)	92.0 (26.7)	(10–35% of daily calories)
Total fat (g)	86.5 (18.3)	(20–35% of daily calories)
Saturated fat (g)	21.0 (8.7)	ND
Monounsaturated fat (g)	34.4 (10.4)	ND
Polyunsaturated fat (g)	23.8 (6.6)	ND
Omega-3 fat (g)	3.4 (2.1)	ND
Trans fat (g)	1.5 (1.4)	ND
Cholesterol (mg)	263.3 (149.2)	ND
**Micronutrients**		
Calcium (mg)	1046.5 (281.2)	[1000]
Sodium (mg)	3064.2 (1201.2)*	[1500]
Iron (mg)	18.2 (4.8)	[27]
Potassium (mg)	3564.4 (1349.1)	[2900]
Magnesium (mg)	439.8 (212.8)	[350]
Phosphorus (mg)	1550.9 (388.5)	[700]
Zinc (mg)	11.9 (2.8)	[11]
Selenium (mcg)	141.6 (46.6)	[60]
Copper (mg)	1.88 (0.9)	[1.0]
Manganese (mg)	6.4 (3.1)	[2.0]
**Vitamins**		
Vitamin C (mg)	145.6 (111.1)	[85]
Vitamin D (mcg)	5.5 (3.4)*	[15]
Vitamin E (mg)	15.4 (7.9)	[15]
Vitamin K (mcg)	418.7 (461.7)	[90]
Total folate (mcg)	553.6 (204.6)*	[600]
Thiamin (B1) (mg)	2.1 (0.5)	[1.4]
Riboflavin (B2) (mg)	2.2 (0.5)	[1.4]
Niacin (B3) (mg)	26.6 (8.9)	[18]
Vitamin B6 (mg)	2.4 (0.8)	[1.9]
Vitamin B12 (mcg)	4.5 (2.6)	[2.6]
Pantothenic acid (mg)	6.2 (1.6)	[6]

*Note* DRIs were obtained from the National Institutes of Health (NIH) Office of Dietary Supplements [32] which was modified from *Dietary Reference Intakes for Energy, Carbohydrate, Fiber, Fat, Fatty Acids, Cholesterol, Protein, and Amino Acids* (2002/2005) and *Dietary Reference Intakes for Water, Potassium, Sodium, Chloride, and Sulfate* (2005). ND: “Not Determined” according to NIH Nutrient Recommendations. Intake values in square brackets indicate DRI’s specific for pregnancy. A “*” indicates that the average intake value is out of range

Ten of the 23 possible Diet Types/Patterns were observed in our sample. Detailed descriptions of Diet Types/Patterns are provided elsewhere [see Additional file 1] [20]. In order of frequency, these patterns were: Flexitarian (*n* = 29; mean HEI = 72), American (*n* = 20; mean HEI = 57), Low-Fat (*n* = 8; mean HEI = 73), Southern (*n* = 8; mean HEI = 62), Mediterranean (*n* = 6; mean HEI = 77), Low-Carb (*n* = 4; mean HEI = 76), No Red Meat (*n* = 4; mean HEI = 84), Vegan (*n* = 3; mean HEI = 69), Vegetarian (*n* = 1; HEI = 87), and Pescatarian (*n* = 1; HEI = 69). Mean diet quality, assessed by the HEI, was 68 (range 12–98). Of the 84 participants, 70 rated the accuracy of their individual dietary assessment results, averaging 87% and ranging 47–100%.

Table 2 compares selected nutrient intakes by participant race for those who identified as Black (*n* = 47) or White (*n* = 28). Based on results of the t-test, the HEI score varied significantly (*p* < 0.001) by race, with mean scores (SD) for Black and White being 61 (23.1) and 81 (19) respectively. Estimated caloric intake did not vary significantly by group (*p* = 0.588), with the mean (SD) for Black and White being 2188 (372) and 2234 (303) respectively.

**Table 2:** Intake of selected nutrients during pregnancy and the healthy eating index (HEI) according to DietID™ compared by black and white race in the prospective birth cohort, research enterprise to advance children’s health (REACH) (*n* = 75 pregnant persons)

Nutrients	Black race	White race	df	t	p-value
	Mean (SD)	Mean (SD)			
HEI	61 (23.1)	81 (19.0)	74	3.7	**< 0.001**
Carbohydrates (g)	265.4 (71.0)	287.9 (48.7)	74	1.7	0.910
Protein (g)	89.9 (28.4)	98.9 (22.5)	74	1.2	0.225
Total fat (g)	89.0 (20.6)	82.9 (14.4)	74	− 2.3	**0.022**
Dietary fiber (g)	26.7 (16.2)	41.9 (17.9)	74	3.8	**< 0.001**
Vitamin D (mcg)	5.4 (4.0)	5.5 (2.3)	74	0.2	0.880
Total folate (mcg)	510.8 (199.5)	635.7 (196.4)	74	2.6	**0.011**
Calcium (mg)	1008.2 (266.7)	1150.8 (267.8)	74	2.2	**0.029**
Iron (mg)	17.3 (4.8)	20.0 (4.4)	74	2.5	**0.017**
Omega-3 Fat (g)	3.1 (1.9)	3.8 (2.3)	74	1.4	0.156

*Note* Of the 84 participants in the dataset, 9 did not identify as Black (*n* = 47) or White (*n* = 28), and therefore are not included here

## Discussion

Here, we describe preliminary dietary intake results using DietID™ for dietary assessment during pregnancy. Dietary assessment with data output including nutrient intakes, dietary patterns, and diet quality can be ascertained quickly. Overall results from our sample of pregnant persons residing in or near Detroit, MI, included inadequate intakes for folate and vitamin D and excessive intake of sodium, with differences in diet quality by race. On average, participants rated the accuracy of their DietID™ results favorably. The aim in presenting these preliminary results is to encourage other researchers to explore the use of novel dietary assessment methods that minimize patient or participant burden.

Our findings are concordant with other studies assessing nutrient intake during pregnancy [5, 6]. A recent study of over 1,000 pregnant persons in the US, found that almost all consumed excess sodium and did not consume enough key nutrients such as folate and vitamin D, among others [31]. Moreover, similar to our findings, a systematic review and meta-analysis of micronutrient intakes during pregnancy in developed countries, showed that folate and vitamin D intakes were consistently below nutrient recommendations [33]. Epidemiological studies of population subgroups, such as pregnant people, should continue to explore and report nutrient intakes to assess the relationship of nutrient intakes with maternal and offspring health outcomes. The DietID™ tool, although a self-report measure, may be a way to decrease random and systematic measurement error, however, future research is necessary.

The “Flexitarian” and “Standard American” dietary patterns were the most prevalent in our study population. The Flexitarian pattern includes mostly vegetarian and sometimes including meat, fish, and/or poultry whereas the Standard American pattern includes highly processed foods, beverages and ingredients [20]. In recent systematic reviews on dietary patterns before/during pregnancy and maternal and birth outcomes, evidence suggests that dietary patterns that are lower in red and processed meats and higher in vegetables, fruits, whole grains, nuts, legumes, and fish are associated with better birth outcomes (e.g., appropriate gestational age and weight at birth) [34]. However, most of the research was conducted in healthy, White women with access to health care [34]. Thus, continuing to explore dietary patterns in racially/ethnically diverse pregnant populations is warranted.

The average HEI found in our population (68) is similar to other studies reporting HEI during pregnancy in the US (e.g., 67.2) [35]. The HEI is useful in providing a composite measure of dietary intake and provides comprehensive information when assessed in combination with nutrient information. Additionally, our findings suggest that participants felt that DietID™ provided an accurate individual dietary assessment. These results are comparative to a recent case study published by DietID™ where non-pregnant participants (*N* = 18) rated the accuracy of their individual dietary assessment to be, on average, 93% [36].

We also show preliminary results comparing overall diet quality and specific nutrient intakes across racial groups. We presented these results for illustrative purposes only. Although our sample is too small to conduct further analyses, our results are similar to previous reports. Compared to White women, Black women had lower overall diet quality in selected populations including those residing on the East coast of the US and WIC recipients in the Southern US [37, 38], lending credibility to our preliminary findings and highlighting a potential area for future efforts to find effective ways to support Black families.

To our knowledge, this is the first study to use DietID™ during pregnancy. A strength of this study was the participation of a diverse sample, with the majority of pregnant persons self-reporting Black race, a group that is often underrepresented in research. Another strength of the sample used here is that participants were eligible regardless of risk level and/or chronic disease status. This factor increases the potential generalizability in future directions since this sample more closely resembles pregnant persons in the US than a high-risk exclusion criteria population would allow. Given that this dietary assessment method is being administered in the ongoing REACH cohort at multiple timepoints, completion rates by commonly underrepresented pregnant populations is a future direction we hope to explore. As our REACH cohort sample increases, we will assess the validity of the DQPN® in our sample using established methods including use of nutrient biomarkers found in blood, urine, and tissues [10].

### Limitations

- A small sample size (*n* = 84) and a single timepoint during pregnancy make it impossible for continuous assessment and evaluation in diet over the course of pregnancy.
- The specific survey completion time could not be determined in this study, as the time that was captured incluced the time participants were logged into DietID™ which is inclusive of the time it takes to complete the screening questions, the survey, and viewing results.
- Due to a small sample size of those who identified as races other than Black and White, we could not compare nutrient intake across additional race categories,
- Diet ID™ does not account for dietary supplements, therefore, intake of some nutrients may be higher than recorded because of supplementation– specifically during pregnancy when many may be taking prenatal vitamins.
- Desirability or response bias among the participants as Diet ID™ was utilized as a self-assessment with limited supervision.

### Electronic supplementary material

Below is the link to the electronic supplementary material.

**Supplementary Material 1:** DietID^TM^’s Dietary Patterns (as of 01-11-2024)

## Data Availability

The datasets used and/or analyzed during the current study are available from the corresponding author on reasonable request.

## Abbreviations

DQPN®: Diet Quality Photo Navigation
REACH: Research Enterprise to Advance Children’s Health
SD: Standard deviation
HEI: Healthy Eating Index
US: United States
FFQ: Food Frequency Questionnaire
ASA-24: Automated Self-Administered 24-hour Dietary Assessment
HFH: Henry Ford Health
EMR: electronic medical records
DRI: Dietary Reference Intakes

## References

[1] Abu-Saad K, Fraser D. Maternal nutrition and birth outcomes. Epidemiol Rev. 2010;32(1):5–25. 10.1093/epirev/mxq001. 10.1093/epirev/mxq001 20237078

[2] Chia A-R, Chen L-W, Lai JS (2019). Maternal dietary patterns and birth outcomes: a systematic review and Meta-analysis. Adv Nutr.

[3] da Mota Santana J, de Oliveira Queiroz VA, Pereira M et al. Associations between maternal dietary patterns and infant birth weight in the NISAMI cohort: a structural equation modeling analysis. Nutrients. 2021;13(11):4054. 10.3390/nu13114054. 10.3390/nu13114054 PMC8623182 34836305

[4] Prado EL, Dewey KG (2014). Nutrition and brain development in early life. Nutr Rev.

[5] Sauder KA, Harte RN, Ringham BM (2021). Disparities in risks of inadequate and excessive intake of micronutrients during pregnancy. J Nutr.

[6] Sauder KA, Cohen CC, Mueller NT (2023). Identifying Foods that optimize intake of key micronutrients during pregnancy. J Nutr.

[7] Savard C, Lemieux S, Carbonneau É et al. Trimester-specific assessment of diet quality in a sample of Canadian pregnant women. Int J Environ Res Public Health. 2019;16(3):311. 10.3390/ijerph16030311. 10.3390/ijerph16030311 PMC6388152 30678329

[8] Bragg MG, Westlake M, Alshawabkeh AN, et al. Opportunities for examining child health impacts of early-life nutrition in the ECHO Program: maternal and child dietary intake data from pregnancy to adolescence. Curr Dev Nutr. 2023:102019. 10.1016/j.cdnut.2023.102019 PMC10681943 38035205

[9] Thompson FE, Subar AF. Chapter 1: Dietary assessment methodology. In: Coulston AM, Boushey CJ, Ferruzzi MG, Delahanty LM, eds. Nutrition in the prevention and treatment of disease (Fourth Edition). Academic Press; 2017:5–48.

[10] Savard C, Lemieux S, Lafrenière J, Laramée C, Robitaille J, Morisset A-S. Validation of a self-administered web-based 24-hour dietary recall among pregnant women. BMC Pregnancy Childbirth. 2018;18:112. 10.1186/s12884-018-1741-1. 10.1186/s12884-018-1741-1 PMC5913813 29685127

[11] Burke LE, Conroy MB, Sereika SM (2011). The effect of electronic self-monitoring on weight loss and dietary intake: a randomized behavioral weight loss trial. Obesity.

[12] Calfas KJ, Sallis JF, Zabinski MF (2002). Preliminary evaluation of a multicomponent program for nutrition and physical activity change in primary care: PACE + for adults. Prev Med.

[13] Six BL, Schap TE, Zhu FM (2010). Evidence-based development of a mobile telephone food record. J Am Diet Assoc.

[14] Ho DKN, Tseng S-H, Wu M-C (2020). Validity of image-based dietary assessment methods: a systematic review and meta-analysis. Clin Nutr.

[15] Eicher-Miller HA, Prapkree L, Palacios C (2021). Expanding the capabilities of nutrition research and health promotion through mobile-based applications. Adv Nutr.

[16] Ji Y, Plourde H, Bouzo V, Kilgour RD, Cohen TR. Validity and usability of a smartphone image-based dietary assessment App compared to 3-day food diaries in assessing dietary intake among Canadian adults: randomized controlled trial. JMIR mHealth uHealth. 2020;8(9):e16953. 10.2196/16953. 10.2196/16953 PMC7511869 32902389

[17] Yaroch AL, Resnicow K, Davis M, Davis A, Smith M, Khan LK. Development of a modified picture-sort food frequency questionnaire administered to low-income, overweight, African-American adolescent girls. J Am Dietetic Assoc. 2000;100(9):1050–1056. 10.1016/S0002-8223(00)00306-0. 10.1016/S0002-8223(00)00306-0 11019353

[18] Katz D, Rhee L, Katz C (2020). Dietary assessment can be based on pattern recognition rather than recall. Med Hypotheses.

[19] DietID. The Science: Background. 2022. Accessed June 11, 2022; 2022. https://www.dietid.com/the-science.

[20] DietID, Diet ID’S Dietary, Patterns. 2022. Accessed July 21 2022; 2022. https://www.dietid.com/goal-diets.

[21] Bernstein A, Moore R, Rhee L, Aronson D, Katz D. A digital dietary assessment tool may help identify malnutrition and nutritional deficiencies in hospitalized patients. Res Ideas Outcomes. 2021;7:e70642. 10.3897/rio.7.e70642.

[22] Turner-Mcgrievy G, Hutto B, Bernhart JA, Wilson MJ. Comparison of the diet ID platform to the automated self-administered 24-hour (ASA24) dietary assessment tool for assessment of dietary intake. J Am Nutr Assoc. 2022;41(4):360–382. 10.1080/07315724.2021.1887775. 10.1080/07315724.2021.1887775 PMC8634522 33705267

[23] Bernstein AM, Rhee LQ, Njike VY, Katz DL (2023). Dietary Assessment by Pattern Recognition: a comparative analysis. Curr Developments Nutr.

[24] Radtke MD, Chodur GM, Bissell MC (2023). Validation of diet ID™ in predicting nutrient intake compared to dietary recalls, skin carotenoid scores, and plasma carotenoids in University students. Nutrients.

[25] Subar AF, Kirkpatrick SI, Mittl B (2012). The automated self-administered 24-hour dietary recall (ASA24): a resource for researchers, clinicians and educators from the National Cancer Institute. J Acad Nutr Dietetics.

[26] PA Harris, R Taylor, R Thielke, J Payne, N Gonzalez, JG. Conde, Research electronic data capture (REDCap) – A metadata-driven methodology and workflow process for providing translational research informatics support. J Biomed Inform. 2009;42(2):377–81. https://pubmed.ncbi.nlm.nih.gov/18929686/10.1016/j.jbi.2008.08.010 PMC2700030 18929686

[27] PA Harris, R Taylor, BL Minor, V Elliott, M Fernandez, L O’Neal, L McLeod, G Delacqua, F Delacqua, J Kirby, SN Duda, REDCap Consortium. The REDCap consortium: Building an international community of software partners. J Biomed Inform. 2019. 10.1016/j.jbi.2019.103208 10.1016/j.jbi.2019.103208 PMC7254481 31078660

[28] Krebs-Smith SM, Pannucci TE, Subar AF (2018). Update of the healthy eating index: HEI-2015. J Acad Nutr Dietetics.

[29] Guenther PM, Kirkpatrick SI, Reedy J (2014). The healthy eating Index-2010 is a valid and reliable measure of diet quality according to the 2010 Dietary guidelines for americans. J Nutr.

[30] Guenther PM, Casavale KO, Reedy J (2013). Update of the healthy eating index: HEI-2010. J Acad Nutr Dietetics.

[31] Bailey RL, Pac SG, Fulgoni VL, Reidy KC, Catalano PM (2019). Estimation of total usual dietary intakes of pregnant women in the United States. JAMA Netw open.

[32] Supplements NIoHNOoD. Nutrient Recommendations: Dietary Reference Intakes (DRI). Accessed June 20, 2022; 2022. https://ods.od.nih.gov/HealthInformation/Dietary_Reference_Intakes.aspx.

[33] Blumfield ML, Hure AJ, Macdonald-Wicks L, Smith R, Collins CE (2013). A systematic review and meta‐analysis of micronutrient intakes during pregnancy in developed countries. Nutr Rev.

[34] Raghavan R, Dreibelbis C, Kingshipp BL (2019). Dietary patterns before and during pregnancy and birth outcomes: a systematic review. Am J Clin Nutr.

[35] Tahir MJ, Haapala JL, Foster LP (2019). Higher maternal diet quality during pregnancy and lactation is associated with lower infant weight-for-length, body fat percent, and fat mass in early postnatal life. Nutrients.

[36] Aronson D. Case study: spectrum health achieves outstanding results with digital lifestyle medicine. Accessed June 6, 2021. https://static1.squarespace.com/static/5d38ca4174e198000123709a/t/60ac16b23fcd080c882d0ce5/1621890739003/spectrum+case+study+printable.pdf.

[37] Bodnar LM, Simhan HN, Parker CB (2017). Racial or ethnic and socioeconomic inequalities in adherence to National Dietary Guidance in a large cohort of US pregnant women. J Acad Nutr Dietetics.

[38] Hill AM, Nunnery DL, Ammerman A, Dharod JM (2020). Racial/ethnic differences in diet quality and eating habits among WIC pregnant women: implications for policy and practice. Am J Health Promotion.

