# Supplementary material for: Exploring preliminary dietary intake results using a novel dietary assessment tool with pregnant participants enrolled in a birth cohort
Source: BMC Res Notes. 2024 Feb 1;17:42. doi: 10.1186/s13104-024-06697-9 (PMC10835830; doi:10.1186/s13104-024-06697-9)
Supplement: Supplementary file 1 — Supplementary Material 1: DietIDTM’s Dietary Patterns (as of 01-11-2024) [file 13104_2024_6697_MOESM1_ESM.docx]

| *DietID™’s Dietary Patterns (as of 01-11-2024)* | |
| --- | --- |
| **Diet Pattern** | **Description** |
| Paleo | Includes a variety of meats (e.g. grass-fed, game), wild fish and seafood, free-range eggs, whole fruits and vegetables, nuts and seeds, with or without non-dairy milk. Limits legumes, grains, dairy products, refined sugars and added salt. |
| Low Carb* | Includes lean meats, poultry, seafood, eggs, mostly non-starchy vegetables, whole fruits, nuts and seeds, variety of fats, with or without dairy/non-dairy products. Limits grains, legumes, and added sugars. |
| Low Fat* | Includes lean meats, poultry and fish, fruits and vegetables, grains, legumes, and low-fat dairy products. May include low-fat processed foods and limited amounts of nuts, seeds, nut butters, olives, avocado, cooking oils, fatty fish, and eggs. |
| No Red Meat* | Includes fruits, vegetables, grains, beans, nuts, seeds, dairy products, eggs, poultry, and fish. May include highly processed foods, beverages, and ingredients. |
| Flexitarian* | Mostly vegetarian diet that sometimes includes meat, fish, and/or poultry. May include highly processed foods, beverages, and ingredients. |
| Vegetarian* | Includes vegetables, fruits, grains, legumes, nuts, seeds, dairy products, and eggs. May include highly processed dairy and plant-based foods. Excludes all other animal products. |
| Vegan* | Comprised of 100% plant-based foods including vegetables, fruits, grains, legumes, nuts, and seeds, as well as processed foods such as baked goods, soyfoods, oils, dairy substitutes, meat substitutes, and sweets/desserts made without dairy or egg. Excludes all animal products. |
| Whole Foods Plant Based | The Whole Foods Plant Based diet includes vegetables, fruits, intact whole grains, nuts, seeds, and legumes, with minimal processed foods (such as soyfoods, whole grain flour-based products) and no added oils, concentrated sugars, or concentrated fats. |
| Whole Foods Plant Based Low Fat | The WFPB Fat-Restricted diet includes vegetables, fruits, intact whole grains, and legumes. It includes with minimal amounts of processed foods (such as soyfoods, whole grain flour-based products), fatty foods (such as avocado and coconut), and nuts and seeds, and has no added oils, concentrated sugars, or concentrated fats. |
| Pescatarian * | Includes seafood (fin fish and shellfish), vegetables, fruits, grains, legumes, nuts, seeds, dairy products, and eggs. May include highly processed dairy and plant-based foods. Excludes all other animal products. |
| Popular American * | Includes fruits, vegetables, grains, beans, nuts, seeds, dairy products, eggs, meats, poultry, and fish. May include highly processed foods, beverages, and ingredients. |
| Mediterranean* | This diet pattern includes vegetables, fruits, nuts and seeds, whole grains, legumes, dairy products, seafood and lean poultry. Emphasis on olive oil, herbs, spices, and red wine (moderation). |
| Mexican-American | The Mexican-American dietary pattern, reflective of the traditional cuisine of Mexico, is based heavily on corn/maize, chile, and beans. The dietary pattern typically includes wheat and other grains, meats, poultry, fish/seafood, eggs, fruits, vegetables, beans, nuts, seeds, sweeteners, and dairy products. May include highly processed foods, beverages and ingredients. |
| Southern American* | Reflective of the style of cuisine common to the Southern states of America, this diet includes fruits, vegetables, grains, beans, nuts, seeds, dairy products, eggs, meats, poultry, and fish. May include highly processed foods, beverages, and ingredients. |
| Caribbean (West Indies) | This diet pattern is based on the traditional cuisines of Trinidad, Jamaica, Guyana, Tobago, and Haiti. There is emphasis on soups and stews, root vegetables, rice, bread, coconut products, tropical fruits, fish, and cow’s milk. Traditionally, the mid-day meal (“lunch”) is the main meal, while the evening meal (“dinner”) consists of lighter fare. |
| South Asian | This dietary pattern is based on Indian cuisine with an emphasis on flatbreads (e.g. roti, chapati, naan), rice, legumes (e.g. chickpeas, lentils, peas), vegetables (e.g. spinach, potatoes, cauliflower), fruits (e.g. mango, banana, papaya), dairy products (e.g. yogurt, milk, paneer), nuts (e.g. peanuts, cashews), eggs, meats, and seafood. Use of whole and ground spices (e.g. cumin, turmeric, chili, asafoetida), fresh herbs (e.g. coriander, mint), and hot chili peppers are commonly added for flavor. |
| South Asian Vegetarian | This dietary pattern is based on Indian cuisine with an emphasis on flatbreads (e.g. roti, chapati, naan), rice, legumes (e.g. chickpeas, lentils, peas), Vegetables (e.g. spinach, potatoes, cauliflower), fruits (e.g. mango, banana, papaya), dairy products (e.g. yogurt, milk, paneer), nuts (e.g. peanuts, cashews), and in some instances, eggs. Use of whole and ground spices (e.g. cumin , turmeric, chilli, asafoetida), fresh herbs (e.g. coriander, mint), and hot chili peppers are commonly added for flavor. |
| Continental European (South) | The Southern European dietary pattern is based on the cuisines common in the countries of Italy, France, Spain, Belgium and Switzerland. |
| Continental European (North) | The Northern European dietary pattern is based on the cuisines common in the countries of Sweden, Norway, Denmark, and Finland. |
| Dash | DASH (Dietary Approaches to Stop Hypertension) includes vegetables, fruits, whole grains, nuts/seeds, legumes, mostly non-fat dairy products, lean meats, poultry and fish, non-tropical vegetable oils. Emphasis is on low-sodium foods. |
| Mind | Mediterranean-DASH Intervention for Neurodegenerative Delay, or MIND, is a therapeutic plan designed to promote a healthy brain and reduce the risk of Alzheimer’s disease. It encourages vegetables, especially leafy greens, fruits, especially berries, nuts, beans, whole grains, fish, poultry, olive oil, and wine. in moderation (if consumed). Saturated fats, fast/fried foods, red meats, cheese, and sweets are discouraged. |
| American Heart Association | This diet includes vegetables, fruits, whole grains, low-fat or non-fat dairy products, lean poultry, fish, egg whites, and non-tropical vegetable oils. Emphasis is on oats, barley, beans, lentils, peas, nuts and seeds. |
| Therapeutic Lifestyle Changes | The Therapeutic Lifestyle Changes (TLC) diet includes vegetables, fruits, whole grains, low-fat or non-fat dairy products, lean poultry, fish, egg whites, and non-tropical vegetable oils. Emphasis is on oats, barley, beans, lentils, peas, nuts and seeds. |
| *Notes.* *indicates diet pattern was present in the current study. Dietary pattern names and descriptions were retrieved 01-11-2024, directly from DietID™’s website (<https://www.dietid.com/goal-diets>). | |
